# Laryngeal extra-skeletal Ewing sarcoma treated with DC-CTL immunotherapy: A case report and review of the literature

**DOI:** 10.3389/fonc.2022.1003393

**Published:** 2022-11-30

**Authors:** Hanrui Wang, Jianwei Wang, Qiang Wang, Yujuan Yang, Jing Guo, Chao Ren, Yakui Mou, Chuanliang Jia, Xicheng Song

**Affiliations:** ^1^ Department of Otorhinolaryngology, Head and Neck Surgery, Yantai Yuhuangding Hospital, Qingdao University, Yantai, China; ^2^ Shandong Provincial Clinical Research Center for Otorhinolaryngologic Diseases, Yantai, China; ^3^ Shandong Provincial Innovation and Practice Base for Postdoctors, Yantai Yuhuangding Hospital, Yantai, China

**Keywords:** larynx, surgery, prognosis, immunotherapy, extra-skeletal Ewing sarcoma

## Abstract

Extra-skeletal Ewing sarcoma (EES) is a rare sarcoma composed primarily of small round cells, capable of metastasizing and relapsing. Few cases of EES originating from the larynx have been reported, and no publications regarding laryngeal EES treated with dendritic cells-cytotoxic T lymphocytes (DC-CTL) immunotherapy have been found. We described a 29-year-old woman with a mass found in the larynx. Diffuse small round cells with scanty cytoplasm shown by histology test and extremely positive staining of CD99 revealed by immunohistochemistry helped determine the diagnosis of laryngeal EES. The patient survived for seven years with no signs of recurrence or metastasis after six cycles of DC-CTL immunotherapy based on traditional treatments. This case indicates that DC-CTL immunotherapy could be considered a new option for treating EES.

## Introduction

Extra-skeletal Ewing sarcoma (EES) is a rare malignancy primarily composed of small round cells (1). A majority of cases are found in adolescents and young adults (2, 3). Most commonly EES affects the soft tissues in the paravertebral region, lungs, kidneys and bladder, with the head and neck region being a rare origin site (1, 4). Patients with EES commonly complain of local masses with or without regional swelling pain, increased skin temperature, and restricted movement of limbs due to nerve invasion (4). However, these symptoms are atypical and difficult to distinguish from other types of malignancies at first consultations. Generally, patients with EES have a poor prognosis and a high risk of metastasis or recurrence after traditional implementation of surgical resection, and postoperative radiotherapy and chemotherapy (5–7). A previous study involving 18 patients found that the 1-year, 3-year, and 5-year survival rates of EES after surgery combined with other treatment modalities were 82.4%, 64.2%, and 32.1%, respectively (8). In order to reach a 5-year survival rate of 60-70%, a combined treatment strategy, including surgery, radiotherapy, and high-dose chemotherapy, is required as soon as the sarcoma manifests itself (4, 9).

Head and neck tumors can initiate from the oropharynx, nasopharynx, laryngopharynx, thyroid gland, cervical trachea and cervical esophagus. According to the WHO Pathological Classification (2017), common tumors in the head and neck can be divided into malignant epithelial tumors, neuroendocrine tumors, and benign epithelial tumors, soft tissue tumors, heamatolymphoid tumors, tumors of bone and cartilage, mucosal malignant melanoma, secondary tumors (10). Patients with head and neck tumors suffer from a wide range of symptoms including a lump in the neck, a sore throat that is difficult to relieve, difficulty swallowing, and hoarseness of voice (11). Some patients similar to this case have only local masses. The late stages of carcinomas or other aggressive malignancies in the head and neck can also result in swelling metastatic lymph nodes (12). As a result, it may be difficult to determine the origin of the mass and its symptoms. In this study, we reported a case of EES with a local mass in the neck as the primary reason for consultation. Because of a lack of specificity in clinical manifestations and subsequent delayed in diagnosis and treatment, patients with EES are likely to have a poor prognosis because the sarcoma has commonly advanced to a late stage (13–15). Here, we described a case of EES which developed from the larynx and was treated with a newly developed biological immunological therapy based on surgery combined with radiotherapy and chemotherapy treatment, with a survival period of more than 5 years.

## Case presentation

**Figure 1:**
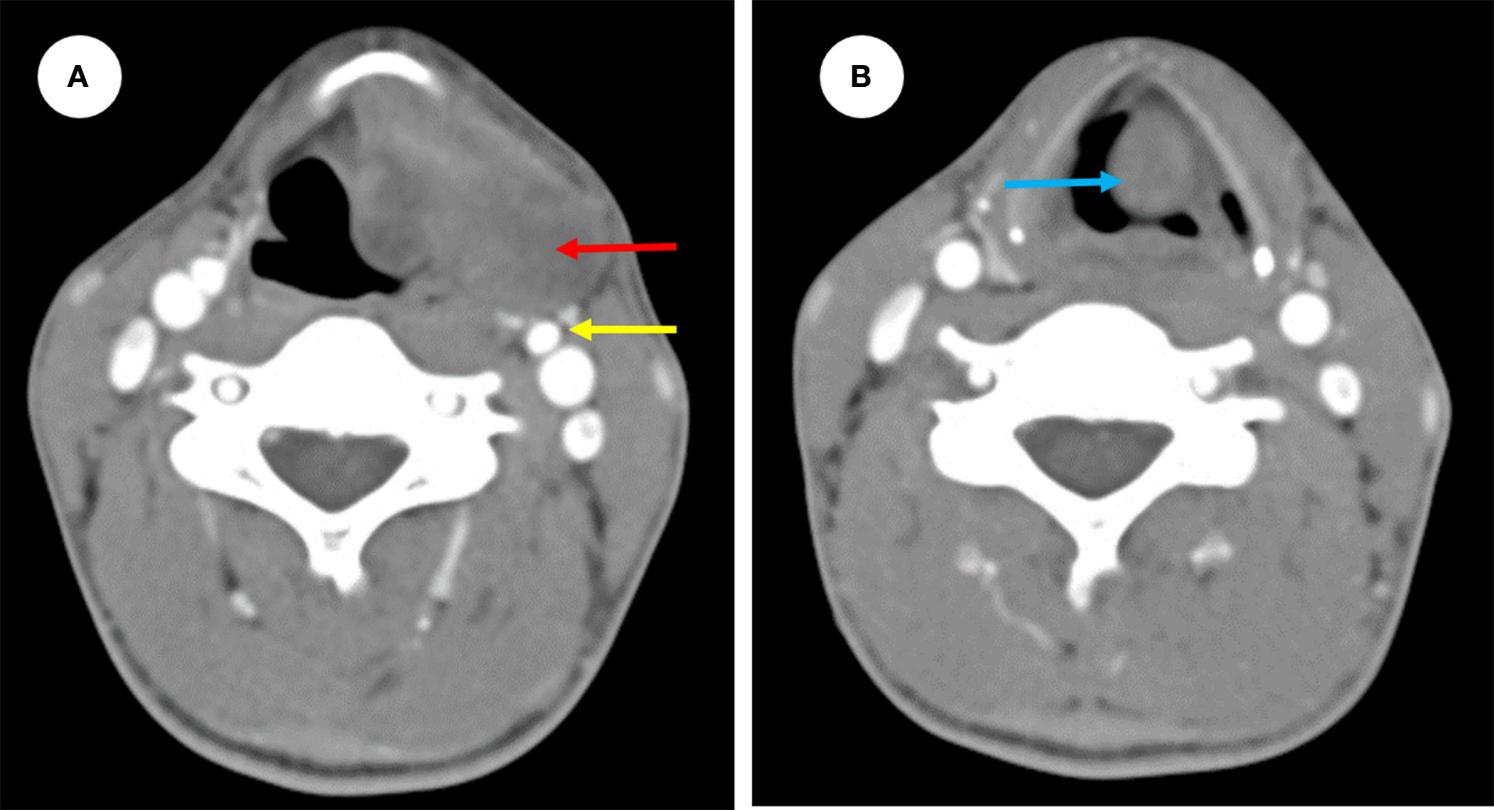
Preoperative enhanced CT images showing a mass of irregular density (red arrow) located anterior to the carotid (yellow arrow) extending into both the left side of preepiglottic space (red arrow) **(A)** and the laryngeal cavity (blue arrow) **(B)**.

**Figure 2:**
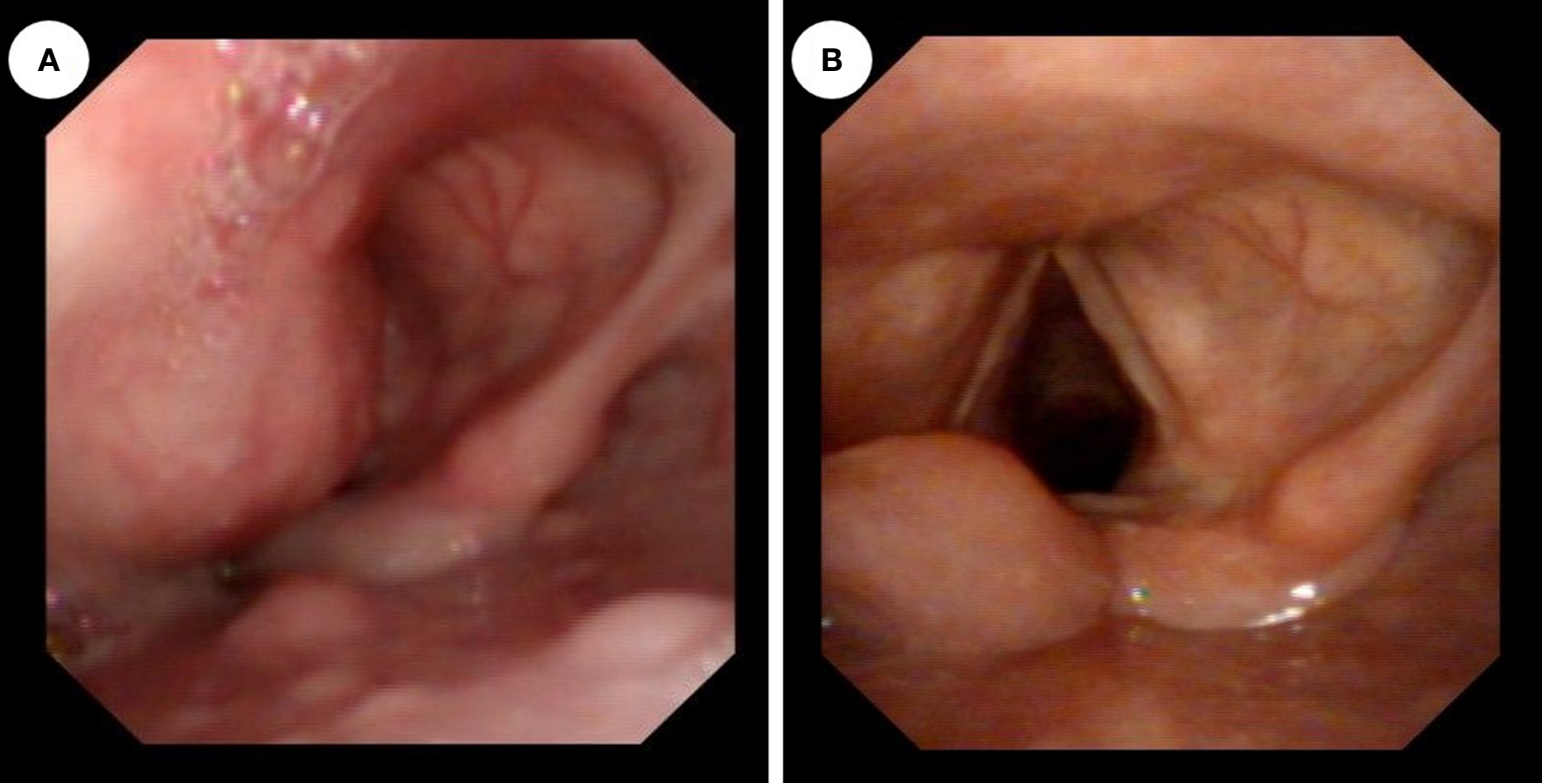
**(A)** Preoperative fiberoptic laryngoscopy showed a smooth bulge in the left aryepiglottic fold, protruding into the laryngeal cavity, and the glottic fissure was invisible. **(B)** After the operation, normal laryngeal structures were observed using a fiberoptic laryngoscope, the glottic region was fully exposed, and there were no obvious abnormalities in the hypopharynx.

In a 29-year-old female, a painless mass in the left neck region had slowly grown over the past two months. The patient denied experiencing dyspnea, dysphagia, laryngalgia, hoarseness, or any other oppression sensations. Furthermore, she had no family history of malignant tumors. Upon physical examination a hard mass in the left submandibular region, approximately 3 cm by 2 cm in size, with normal skin temperature and color and good mobility. There were no enlarged lymph node or thyroid mass found. Laboratory examinations revealed no abnormality. An ultrasound-guided pathological biopsy of the tumor reveal that it was malignant. Afterward, enhanced computed tomography (CT) showed a mass of irregular density located anterior to the carotid and extending into both the laryngeal cavity and the left piriform fossa (**Figure 1**). The mass was found to be lobulated, with a maximum cross-sectional area of 3.8 cm by 2.0 cm. A smooth protrusion in the laryngeal cavity was seen under fibro-laryngoscope (**Figure 2A**). Additionally, a PET-CT scan indicated no distant metastasis.

After the malignancy of the tumor was confirmed, surgical excision was performed. During the surgery, an arcuate incision of about 5 cm in length was made along the dermatoglyphic pattern at the bulge of the mass in the left neck. Subsequently, the skin, subcutaneous tissue, and cervical pinna muscles were incised to separate the flaps. The surgical boundary was superior to the level of the hyoid bone and inferior to the level of the left thyroid cartilage notch. After the incision of the deep cervical fascia, a mass of about 3.5cm by 3.0 cm by 2.0 cm was revealed in the left greater horn of the hyoid bone and the left superior cornu of the thyroid cartilage. The tumor was lobulated and slightly firm with intact envelope. The superior laryngeal artery was ligated to protect the superior laryngeal nerve, and the mass was carefully separated along the capsule. It was bluntly separated along the capsule on the surface, and the tumor protruded into the preepiglottic space and paraglottic space across the upper-left edge of the thyroid cartilage and laryngeal mucosa was not involved. Negative pressure drainage was placed in the surgical cavity and the incision was sutured layer by layer. No metastatic lymph node was found.

**Figure 3:**
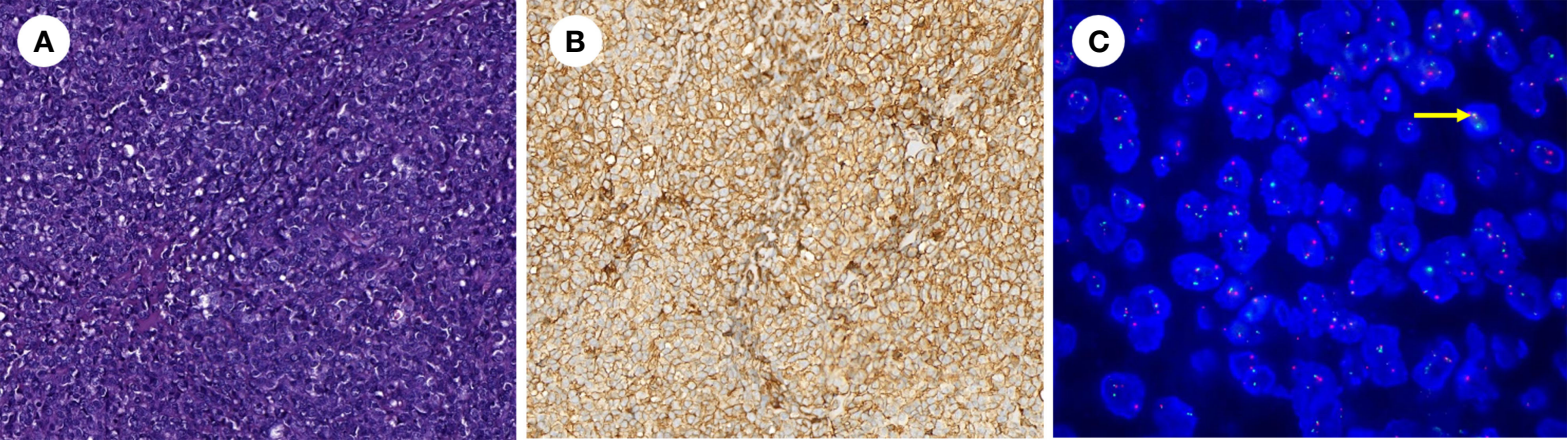
**(A)** Hematoxylin–eosin stain showing the mass consists of small round cells (hematoxylin-eosin, original magnification ×200). **(B)** Immunohistochemistry revealed extremely positive CD99 staining (× 200). **(C)** The fusion of EWSR1 with FLI1 was positively detected by FISH in tumor cells (yellow arrow) (× 1000).

The excised mass was lobulated and hard. Microscopically, it seemed to be nodular in distribution and was composed of diffuse small round or oval cytoplasm deficient cells with marked cellular heterogeneity, evident nuclear division and scattered necrosis (**Figure 3**). Anti-CD99 antibody (CD99) (ZA-0577, ZSGB-BIO, Wuxi, China) immunohistochemistry results showed extremely positive staining (approximately 100%). Furthermore, immunohistochemistry results revealed that calcitonin (CT) (ZA-0578, ZSGB-BIO, Wuxi, China), CD3 (ZA-0503, ZSGB-BIO, Wuxi, China), CD20 (ZM-0039, ZSGB-BIO, Wuxi, China), CD21(ZM-0040, ZSGB-BIO, Wuxi, China), leukocyte common antigen (LCA) (ZM-0183, ZSGB-BIO, Wuxi, China), Melanoma Marker (HMB45) (ZM-0187, ZSGB-BIO, Wuxi, China), S-100 (ZA-0225, ZSGB-BIO, Wuxi, China), and Myogenin (MY) (ZA-0592, ZSGB-BIO, Wuxi, China) were all negative. Moreover, the fusion of Ewing sarcoma breakpoint region 1 (EWSR1) with Friend leukaemia integration-1(FLI1) was detected using EWSR1 and FLI1 probes (F.01253-01, GZLBP, Guangzhou, China) by fluorescence *in situ* hybridization (FISH), and there were positive findings in the tumor cells (**Figure 3C**). Everything mentioned above led to a diagnosis of ESS originating from the larynx.

**Figure 4:**
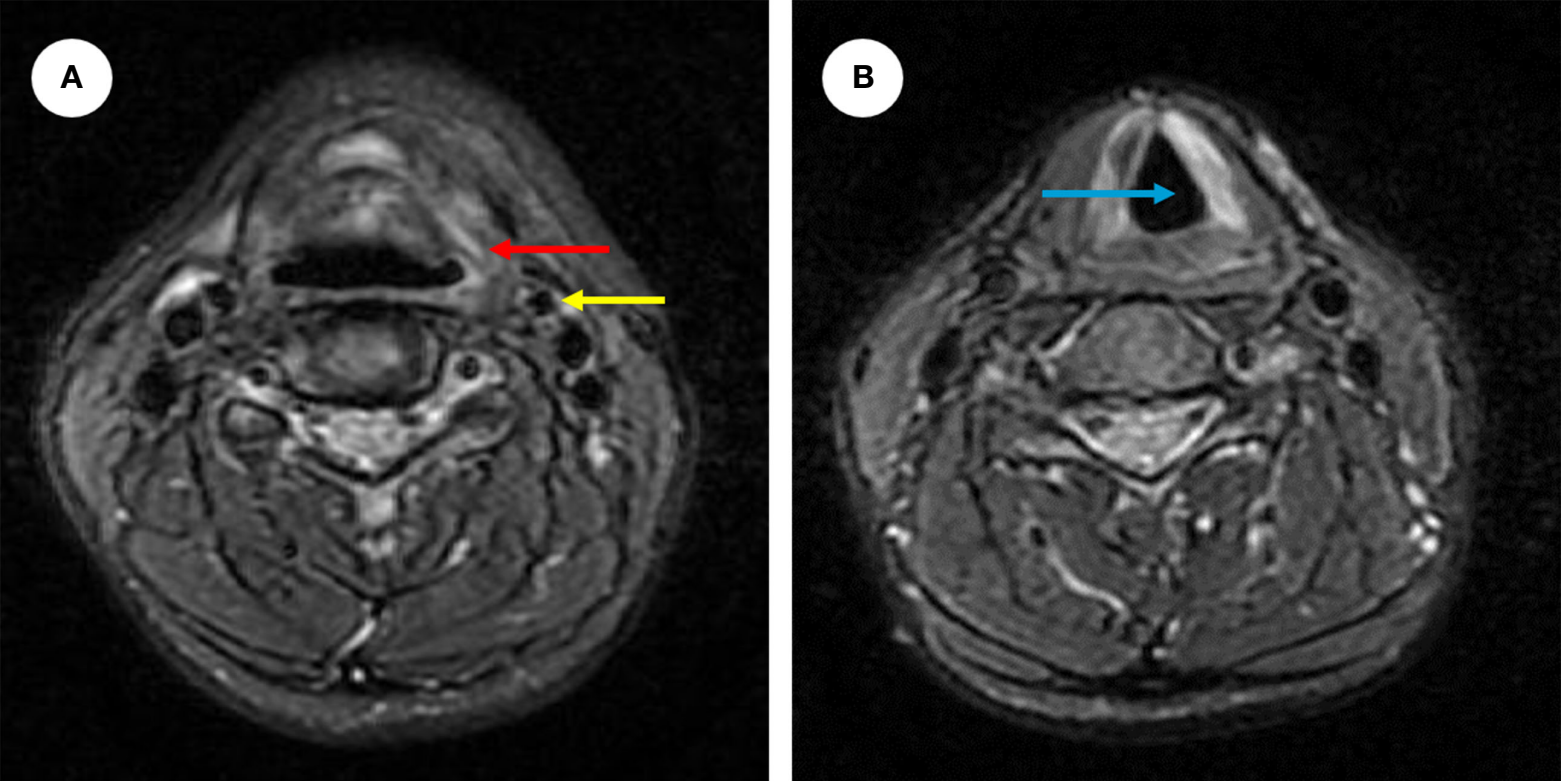
Enhanced MR images showing no tumor recurrence in the precarotid (yellow arrow) and preepiglottic spaces (red arrow) **(A)** and the normal anatomy of the laryngeal cavity (blue arrow) **(B)**.

Postoperatively, after anti-inflammatory treatment and 4 days of surgical cavity drainage, the patients recovered well and a fibro-laryngoscope revealed a healed laryngopharyngeal mucosa. On the tenth day after surgery, the surgical sutures in the neck were removed. The patient did not complain of dyspnea and dysphagia. To reduce the risk of recurrence, postoperative radiotherapy and chemotherapy were performed. Forty days after the operation, the patient underwent 30 times of radiation totaling 60Gy within 6 weeks. And the chemotherapy began 16 days after the radiation, which was 4 cycles of intravenous transfusion of 2.0 grams of isosfamide from day 1 to 5 and 5 milligrams of epirubincin in day 1 or day 1 and 2, and each cycle lasted for 5 days. No adverse event was reported. In addition, the patient accepted cellular immunotherapy considering the rarity of EES and its poor prognosis. The cellular immunotherapy paradigm involved 6 cycles of autotransfusion of immune cells *via* intravenous transfusion, each cycle includes four cell infusions. We collected peripheral blood from patients to culture DC-CTL cells, and DC cells were stimulated with autologous tumor lysates (100 μg/ml) on the 6th day of culture. Then, the DC cells were harvested and co-cultured with CTL on the 7th day and total 14 days were cells cultured until first harvest (The protocol used for generation of DC-CTL in reference 16). The final cell suspension was mentioned as 2200 ml. We harvested 1000 ml of suspension for each day and supplemented them with 700 ml of fresh medium on the day 1 and day 2, 400 ml of fresh medium on the day 3. The proportion of CD3+/CD8+ CTL (Kit: 662967, BD Bioscience, USA) detected by flow cytometry (Canto II, BD Bioscience, USA; DIVA software, BD Bioscience, USA) and the number of cells per reinfusion are as follows: cycle 1, CD3+/CD8+ CTL accounts for 70.50%, day 1 (D1) 1.451×10^9 cells, D2 1.016×10^9 cells, D3 1.312×10^9 cells and D4 1.557×10^9 cells; cycle 2, CD3+/CD8+ CTL accounts for 73.03%, D1 0.7×10^9 cells, D2 1.432×10^9 cells, D3 0.848×10^9 cells and D4 1.336×10^9 cells; cycle 3, CD3+/CD8+ CTL accounts for 61.02%, D1 1.512×10^9 cells, D2 1.24×10^9 cells, D3 1.112×10^9 cells and D4 0.864×10^9 cells; cycle 4, CD3+/CD8+ CTL accounts for 84.51%, D1 1.272×10^9 cells, D2 0.954×10^9 cells, D3 1.16×10^9 cells and D4 0.896×10^9 cells; cycle 5, CD3+/CD8+ CTL accounts for 84.87%, D1 0.8×10^9 cells, D2 0.768×10^9 cells, D3 0.448×10^9 cells and 0.656×10^9 cells; cycle 6, CD3+/CD8+ CTL accounts for 56.50%, 1.44×10^9 cells, 0.984×10^9 cells, 0.72×10^9 cells and 1.504×10^9 cells. Additionally, 100IU of recombinant human interleukin 2 was injected subcutaneously after cell re-infusion on D2 and D4. Laboratory examinations showed no abnormality and the patient reported no discomfort following each immunotherapy session. One year after the combined treatment strategy, enhanced magnetic resonance imaging (MR) revealed no evidence of recurrence (**Figure 4**). A postoperative fiberoptic laryngoscopy revealed normal laryngeal structures (**Figure 2B**). Seven years after surgery, the patient showed no signs of recurrence or metastasis.

## Discussion

EES is a rare sarcoma in the neck region, with rare laryngeal EES reported. Since the discovery of Ewing sarcoma in 1921, few cases of EES, a subset of Ewing sarcoma that developed from extraosseous tissue, have been reported (17). Generally, for patients with EES, surgery combined with radiotherapy and chemotherapy would be strongly recommended, despite a poor prognosis and extremely low 5-year survival rate (6, 18). In this study, we reported a case of EES arising from the larynx, which extended into both the laryngeal cavity and the piriform fossa, and investigated the potential of dendritic cells-cytotoxic T lymphocytes (DC-CTL), an emerging biological therapy, in the treatment of EES.

EES is a highly aggressive malignancy. However, due to the lack of specificity in clinical manifestation, this type of sarcoma is difficult to diagnose. EES, like most malignant tumors, only shows heterogeneously enhanced lesions upon medical imaging, or results in compressive symptoms when the sarcoma grows large (8). Imaging interpretation alone could not exclude a primary thyroid tumor in this patient. Fine needle aspiration pathology also only suggests its malignancy, and in order to identify the tumor surgical resection was required to determine its specific nature.

While microscopic small round cells can alert clinicians of the presence of uncommon tumor in the head and neck, it may be difficult to distinguish the EES from other small round cell tumors such as rhabdomyosarcoma (19). In such a situation, immunohistochemistry would be necessary. In this patient, negative calcitonin (CT) staining ruled out the possibility of medullary thyroid carcinoma (20), the negative staining of CD3, CD20, CD21 and LCA excluded the possibility of lymphoma (21), the HMB45 (-) and S-100 (-) eliminated the chance of melanoma (22), and the MY (-) ruled out the probability of rhabdomyosarcoma (20). Moreover, a highly positive CD99 staining aided in the diagnosis of EES (23). CD99, regulated by EWSR1–FLI1, is a cell-surface glycoprotein and a useful diagnostic marker for Ewing sarcoma (24, 25). In immunochemistry tests, diffuse membranous expression of CD99 is evident and the accuracy can reach 95% in Ewing sarcomas (26).

Traditionally, surgical excision remains the mainstay of EES treatment, with radiotherapy and chemotherapy used as adjuvant empirical therapies (19). To gain a better understanding of laryngeal and cervical EES, previous studies were reviewed and listed in **Table 1** (27–53). The studies are limited to English language literature published between 1982–2021. In the previous 32 cases, 3 tumors originated from the larynx and composed approximately 9.1% cervical EES. We found that 21 of these patients had clear documentation of treatment modalities and prognoses. Further, we compared the effectiveness of different treatment modalities on prognosis finding that the average survival period of patients who underwent surgical excision (n = 15) was 25 months and that those without surgery (n = 6) survived 41.33 months on average. Patients who received radiotherapy (n=15) survived 36.20 months on average, while those who did not receive radiotherapy (n = 6) survived 13.33 months. The survival period of chemotherapy-treated patients (n = 19) was 32.05 months vs. 7.00 months for those who did not receive chemotherapy (n = 2). It is noteworthy that each of these patients accepted at least one kind of conventional modality of treatment. The study indicates that radiotherapy and chemotherapy play a better role in treating cervical EES. However, the assumption is based on a small sample size, which may lead to biased speculation. Furthermore, despite the fact that most patients with cervical EES were treated with combined treatment modalities, only three patients survival more than 5 years. Therefore, the effectiveness of different modalities of EES treatment should be further evaluated through more studies. In addition, patients with cervical EES have a poor prognosis, a high recurrence rate ranging from 15 to 30%, and a low 5-year survival rate (54). Therefore, a more effective treatment to improve EES prognosis is required.

**Table 1:** Summary of previous published articles on cervical EES.

References	No.	Age (years)	Gender	Lesion site	Tumor size	Clinical manifestation	Treatment	Follow-up (months)	Outcome
Ansari MH et al. (2019) (27)	1	3	F	Right-sided neck	4×3×1cm	Right-sided neck swelling for 2 months	S	Unknown	NED
Whaley JT, et al. (2010) (28)	2	19	F	Post neck muscle	Diameter 4cm	Unknown	CT+XRT	156	NED
Abdel Rahman H, et al. (2010) (29)	3-6	Unknown	Unknown	Neck	Unknown	Unknown	Unknown	Unknown	Unknown
Yao-jie FENG, et al. (2020) (30)	7	Unknown	Unknown	Root of neck	Unknown	Unknown	Unknown	Unknown	Unknown
Yao-jie FENG, et al. (2020) (30)	8	Unknown	Unknown	Submandibular	Unknown	Unknown	Unknown	Unknown	Unknown
Yi-liang HOU, et al. (2002) (31)	9	18	M	Root of right-sided neck	1.4×1.0×0.8cm	Movable neck mass with slight pain	S + CT+XRT	36	NED
Ying CHEN, et al. (2002) (32)	10	27	F	Unknown	12×10×8cm	Notable neck mass with tenderness	S + CT+XRT	24	NED
Xiao-long LIN, et al. (2021) (33)	11	30	F	Left-sided neck	8×5cm	Notable mass in the left neck with swelling pain in the throat	S+ CT	9	Lost to follow-up
Shuang Wang, MD, et al. (2021) (34)	12	36	F	Left-sided neck	9×8×6cm	Notable painless mass with dysphagia	S+ CT+XRT	19	NED
Gazula S, et al. (2019) (35)	13	4-month-old	F	Left submandibular	2.9×2.8×2.0cm	Notable swelling for 3 weeks, with the mass increasing gradually	S + CT	24	NED
Ali S, et al. (2008) (36)	14	14	M	Right-sided neck	12×5cm	A rapidly extending mass for 1 month	CT+XRT	Unknown	Unknown
Van Der Meer G, et al. (2017) (37)	15	12	M	Posterior to tracheal and anterior to vertebra	Diameter 3.5cm	Sleep disorder, short of breathing, sore throat and stridor	CT+XRT	18	Radiotherapy causing an edematous larynx and mucositis
Maroun CA, et al. (2019) (38)	16	54	M	Right paraglottic space	5.0×3.8×3.8cm	Notable mass with hoarseness	CT+XRT	12	NED
Yang YS, et al. (2004) (39)	17	74	M	Larynx	3.5×2.0cm	Acute aggravate dyspnea	S +XRT	6	NED
Lynch MC, et al. (2014) (40)	18	45	F	Larynx	Diameter 2.9cm	A rapidly growing lump in the right side of neck with hoarseness.	CT+XRT	Unknown	NED
Wygoda A, et al. (2013) (41)	19	68	M	Larynx	2.0×1.9×1.7cm	Hoarseness and occasional aphonia	CT+XRT	30	NED
Khosla D, et al. (2019) (42)	20	8	F	Parapharyngeal space	Unknown	Difficulty in breathing and swallowing, earache and bleeding from the mouth	CT+XRT	12	Died of distant metastasis
Cho SI, et al. (2007) (43)	21	49	M	The parapharynx with pulmonary metastasis.	Unknown	Diplopia and mild headache	CT	20	No improvement of metastatic lesions in rectum and blindness.
Rama-López J, et al. (2017) (44)	22	70	M	Left supraclavicular fossa	6.1×6.7×7.1cm	A rapidly growing mass	NC+ S + CT+XRT	24	NED
Gustafson RO, et al. (1982) (45)	23	18	M	Right-side neck	14×10×7cm	Notable growing mass	S + CT+XRT	28	NED
Schmidt S, et al. (2010) (46)	24	16	M	Neck	Unknown	Painful lump	S+ CT+XRT	7	NED
Chirila M, et al. (2013) (47)	25	48	M	Thyroid	10×10cm	Thyroid recurrent episodes of acute obstructive respiratory distress	S + CT	1	Died
Adapa P, et al. (2009) (48)	26	9	F	Thyroid	4.0×4.5×6.0cm	Painless swelling	S + CT+XRT	72	NED
Wei-yu ZHU, et al. (2021) (49)	27	30	F	Left supraclavicular region	6×5×3cm	Progressive enlarged mass with feeling of swallowing obstruction	S + CT+XRT	36	NED
Kabata P, et al. (2017) (50)	28	34	M	In the lower left neck	5.8×5.8×6.0cm	Upper respiratory tract infection, sore throat, and difficulties in swallowing	NC + S	18	NED
Bishop JA, et al. (2015) (51)	29	19	M	Thyroid	Unknown	Neck mass	S	Unknown	NED
Bishop JA, et al. (2015) (51)	30	36	F	Thyroid	Unknown	Goiter	S	Unknown	Unknown
Maldi E, et al. (2012) (52)	31	66	M	Thyroid	Diameter 4.5cm	A single nodule on the left lobe of the thyroid gland	S	8	Metastatic disease has been discovered
Seipel AH, et al. (2021) (53)	32	54	F	Thyroid	3.7×3.1×2.1cm	Unknown	S + CT+XRT	15	NED

F, female; M, male; S, Surgery; CT, chemotherapy; NC, Neoadjuvant chemotherapy; XRT, radiotherapy; NED, no evidence of disease; DC-CTL, dendritic cells-cytotoxic T lymphocyte immunotherapy.

Currently, cellular immunotherapy plays an increasingly important role in the treatment of malignancies (55, 56). It could kill tumor cells by extracting immune cells from patients’ peripheral blood and reinfusing them after activation and expansion (57). Dendritic cells (DC), natural killer cells (NK), and cytotoxic T lymphocytes (CTL) could be the cell types extracted and expanded (57–59). Physicians should choose an immune cell type appropriate for the specific carcinoma or sarcoma when using cellular immunotherapy (57). Until now, studies have shown that DC-CTL immunotherapy can improve the prognosis and quality of life of patients with liver cancer, small cell lung cancer, prostate cancer, and other cancers (60, 61). There are also studies showing that hematopoietic stem cell transplantation or immune cell biotherapy can extend the life expectancy of patients with EES to some extent when combined with traditional treatments (62, 63). The patient who we studied received six cycles of dendritic cells-cytotoxic T lymphocytes (DC-CTL) immunotherapy after surgery, as well as radiotherapy and chemotherapy. In the process of DC-CTL treatment, DC could promote the proliferation of CTL and induce tumor-specificity CTL, and the expanded CTL will then promote its binding capacity to the targeted tumor cells, causing tumor cells to dissolve and die (64). However, due to the low incidence of EES, the effectiveness of cellular immunotherapy is not representative or convincing. Furthermore, there is possibility of immune rejection complications which should be monitored. Fortunately, the patient studied survived more than 5 years without recurrences or complications. We have reasons to believe that DC-CTL therapy could be considered an adjuvant therapy for malignancies. To the best of our knowledge, this was the first case of EES arising from the larynx treated with DC-CTL therapy. However, the true contribution of DC-CTL to curing EES remains unclear, due to the traditional treatment that preceded its use. Furthermore, because of our lack of experience, the efficacy of DC-CTL therapy should be evaluated in more malignancies, and the side effects should be appraised simultaneously.

## Conclusions

We described a rare case of laryngeal EES that was successfully treated with traditional surgery, radiotherapy and chemotherapy, as well as newly developed DC-CTL therapy. It provides us with a new treatment option for EES or other malignancies.

## Data availability statement

The original contributions presented in the study are included in the article/supplementary material. Further inquiries can be directed to the corresponding authors.

## Ethics statement

The studies involving human participants were reviewed and approved by Ethical Committee of Yantai Yuhuangding Hospital. The patients/participants provided their written informed consent to participate in this study.

## Author contributions

XS and CJ made the diagnosis; HW and JW wrote the initial draft of the manuscript; XS, QW, and YM provided clinical data; QW, CR, and JW proofread manuscript; HW, JG, and YY provided follow-up information; CJ and CR reviewed the literature and previous research; all the authors took part in the revision of the manuscript and approved the submission.

## Funding

Taishan Scholars Project (ts20190991); Yantai Science and Technology Program (2022YD006).

## Conflict of interest

The authors declare that the research was conducted in the absence of any commercial or financial relationships that could be construed as a potential conflict of interest.

## Publisher’s note

All claims expressed in this article are solely those of the authors and do not necessarily represent those of their affiliated organizations, or those of the publisher, the editors and the reviewers. Any product that may be evaluated in this article, or claim that may be made by its manufacturer, is not guaranteed or endorsed by the publisher.
